# Supplementary material for: Attachment-related perceptions of life events
Source: PLoS One. 2026 Jan 6;21(1):e0340082. doi: 10.1371/journal.pone.0340082 (PMC12774378; doi:10.1371/journal.pone.0340082)
Supplement: S1 Table — (DOCX) [file pone.0340082.s001.docx]

| Supplementary Table S1. Associations between Event Characteristic Descriptives and Attachment Orientations in the Context of Multi-level Modeling | | |
| --- | --- | --- |
|  | beta | |
|  | Attachment Anxiety | Attachment Avoidance |
| Challenge | **0.075** | **-0.069** |
| Worldview | **0.048** | **-0.064** |
| Emotional Significance | **0.060** | **-0.080** |
| Control | 0.003 | 0.022 |
| Extraordinariness | -0.006 | 0.006 |
| Impact | **0.073** | **-0.061** |
| Predictability | **0.027** | -0.020 |
| Social Status | **0.039** | 0.005 |
| Valence | **-0.037** | **0.048** |
| *Note*. Bolded correlations are significant at *p* < .05. | | |
