# Supplementary material for: Attachment-related perceptions of life events
Source: PLoS One. 2026 Jan 6;21(1):e0340082. doi: 10.1371/journal.pone.0340082 (PMC12774378; doi:10.1371/journal.pone.0340082)
Supplement: S2 Table — (DOCX) [file pone.0340082.s002.docx]

| Supplementary Table S2. Associations between attachment orientations and life event perceptions (by life event) | | | | | |  |  |  |  |  |  |  |  |  |
| --- | --- | --- | --- | --- | --- | --- | --- | --- | --- | --- | --- | --- | --- | --- |
| Valence | Event | ECQ Dimension | Attachment Anxiety | | | Attachment Anxiety (Partialled) | | | Attachment Avoidance | | | Attachment Avoidance (Partialled) | | |
|  |  |  | r | 95% CI LB | 95% CI UB | r | 95% CI LB | 95% CI UB | r | 95% CI LB | 95% CI UB | r | 95% CI LB | 95% CI UB |
| Positive | Different Country | Challenge | 0.098 | -0.044 | 0.236 | 0.111 | -0.031 | 0.249 | -0.057 | -0.197 | 0.085 | -0.079 | -0.218 | 0.063 |
| Positive | Different Country | Worldview | 0.170 | 0.029 | 0.304 | 0.192 | 0.052 | 0.325 | -0.101 | -0.239 | 0.041 | -0.133 | -0.270 | 0.009 |
| Positive | Different Country | Emotional Significance | 0.164 | 0.023 | 0.299 | 0.183 | 0.042 | 0.316 | -0.091 | -0.230 | 0.051 | -0.118 | -0.255 | 0.024 |
| Positive | Different Country | Control | 0.069 | -0.073 | 0.209 | 0.063 | -0.079 | 0.203 | 0.040 | -0.102 | 0.181 | 0.028 | -0.114 | 0.169 |
| Positive | Different Country | Extraordinariness | 0.002 | -0.140 | 0.144 | -0.008 | -0.149 | 0.134 | 0.042 | -0.100 | 0.183 | 0.049 | -0.093 | 0.189 |
| Positive | Different Country | Impact | 0.198 | 0.058 | 0.330 | 0.211 | 0.072 | 0.342 | -0.051 | -0.191 | 0.091 | -0.090 | -0.229 | 0.052 |
| Positive | Different Country | Predictability | -0.032 | -0.173 | 0.110 | -0.060 | -0.200 | 0.082 | 0.157 | 0.016 | 0.292 | 0.161 | 0.020 | 0.296 |
| Positive | Different Country | Social Status | -0.135 | -0.271 | 0.007 | -0.151 | -0.286 | -0.010 | 0.062 | -0.080 | 0.202 | 0.094 | -0.048 | 0.233 |
| Positive | Different Country | Valence | 0.105 | -0.037 | 0.243 | 0.123 | -0.019 | 0.260 | -0.091 | -0.230 | 0.051 | -0.108 | -0.246 | 0.034 |
| Positive | Graduated | Challenge | 0.141 | -0.001 | 0.277 | 0.162 | 0.021 | 0.297 | -0.075 | -0.214 | 0.067 | -0.109 | -0.247 | 0.033 |
| Positive | Graduated | Worldview | 0.107 | -0.035 | 0.245 | 0.133 | -0.009 | 0.270 | -0.101 | -0.239 | 0.041 | -0.128 | -0.265 | 0.014 |
| Positive | Graduated | Emotional Significance | 0.065 | -0.077 | 0.205 | 0.094 | -0.048 | 0.233 | -0.116 | -0.253 | 0.026 | -0.134 | -0.270 | 0.008 |
| Positive | Graduated | Control | 0.048 | -0.094 | 0.188 | 0.055 | -0.087 | 0.195 | -0.026 | -0.167 | 0.116 | -0.037 | -0.178 | 0.105 |
| Positive | Graduated | Extraordinariness | 0.007 | -0.135 | 0.148 | -0.012 | -0.153 | 0.130 | 0.089 | -0.053 | 0.228 | 0.090 | -0.052 | 0.229 |
| Positive | Graduated | Impact | 0.108 | -0.034 | 0.246 | 0.114 | -0.028 | 0.252 | -0.014 | -0.155 | 0.128 | -0.039 | -0.180 | 0.103 |
| Positive | Graduated | Predictability | -0.022 | -0.163 | 0.120 | -0.024 | -0.165 | 0.118 | 0.006 | -0.136 | 0.147 | 0.011 | -0.131 | 0.152 |
| Positive | Graduated | Social Status | 0.009 | -0.133 | 0.150 | 0.018 | -0.124 | 0.159 | -0.038 | -0.179 | 0.104 | -0.041 | -0.182 | 0.101 |
| Positive | Graduated | Valence | -0.052 | -0.192 | 0.090 | -0.088 | -0.227 | 0.054 | 0.148 | 0.007 | 0.284 | 0.164 | 0.023 | 0.299 |
| Positive | Marriage | Challenge | 0.183 | 0.042 | 0.316 | 0.154 | 0.013 | 0.289 | 0.162 | 0.021 | 0.297 | 0.127 | -0.015 | 0.264 |
| Positive | Marriage | Worldview | 0.030 | -0.112 | 0.171 | 0.045 | -0.097 | 0.185 | -0.064 | -0.204 | 0.078 | -0.072 | -0.211 | 0.070 |
| Positive | Marriage | Emotional Significance | 0.004 | -0.138 | 0.146 | 0.051 | -0.091 | 0.191 | -0.211 | -0.342 | -0.072 | -0.217 | -0.348 | -0.078 |
| Positive | Marriage | Control | -0.004 | -0.146 | 0.138 | -0.016 | -0.157 | 0.126 | 0.053 | -0.089 | 0.193 | 0.055 | -0.087 | 0.195 |
| Positive | Marriage | Extraordinariness | -0.006 | -0.147 | 0.136 | -0.034 | -0.175 | 0.108 | 0.124 | -0.018 | 0.261 | 0.128 | -0.014 | 0.265 |
| Positive | Marriage | Impact | 0.072 | -0.070 | 0.211 | 0.095 | -0.047 | 0.233 | -0.094 | -0.233 | 0.048 | -0.113 | -0.251 | 0.029 |
| Positive | Marriage | Predictability | -0.020 | -0.161 | 0.122 | -0.032 | -0.173 | 0.110 | 0.051 | -0.091 | 0.191 | 0.057 | -0.085 | 0.197 |
| Positive | Marriage | Social Status | -0.081 | -0.220 | 0.061 | -0.094 | -0.233 | 0.048 | 0.049 | -0.093 | 0.189 | 0.068 | -0.074 | 0.208 |
| Positive | Marriage | Valence | 0.077 | -0.065 | 0.216 | 0.106 | -0.036 | 0.244 | -0.120 | -0.257 | 0.022 | -0.140 | -0.276 | 0.002 |
| Positive | Moved | Challenge | 0.133 | -0.009 | 0.270 | 0.155 | 0.014 | 0.290 | -0.093 | -0.232 | 0.049 | -0.123 | -0.260 | 0.019 |
| Positive | Moved | Worldview | 0.123 | -0.019 | 0.260 | 0.114 | -0.028 | 0.252 | 0.061 | -0.081 | 0.201 | 0.034 | -0.108 | 0.175 |
| Positive | Moved | Emotional Significance | 0.290 | 0.155 | 0.415 | 0.295 | 0.160 | 0.419 | 0.004 | -0.138 | 0.146 | -0.055 | -0.195 | 0.087 |
| Positive | Moved | Control | 0.020 | -0.122 | 0.161 | 0.006 | -0.136 | 0.147 | 0.081 | -0.061 | 0.220 | 0.073 | -0.069 | 0.212 |
| Positive | Moved | Extraordinariness | -0.025 | -0.166 | 0.117 | -0.034 | -0.175 | 0.108 | 0.052 | -0.090 | 0.192 | 0.053 | -0.089 | 0.193 |
| Positive | Moved | Impact | 0.092 | -0.050 | 0.231 | 0.090 | -0.052 | 0.229 | 0.032 | -0.110 | 0.173 | 0.005 | -0.137 | 0.147 |
| Positive | Moved | Predictability | 0.006 | -0.136 | 0.147 | -0.016 | -0.157 | 0.126 | 0.118 | -0.024 | 0.255 | 0.114 | -0.028 | 0.252 |
| Positive | Moved | Social Status | 0.062 | -0.080 | 0.202 | 0.035 | -0.107 | 0.176 | 0.138 | -0.004 | 0.274 | 0.129 | -0.013 | 0.266 |
| Positive | Moved | Valence | -0.119 | -0.256 | 0.023 | -0.119 | -0.256 | 0.023 | 0.006 | -0.136 | 0.147 | 0.014 | -0.128 | 0.155 |
| Positive | New Friend | Challenge | 0.205 | 0.065 | 0.337 | 0.197 | 0.057 | 0.329 | 0.079 | -0.063 | 0.218 | 0.052 | -0.090 | 0.192 |
| Positive | New Friend | Worldview | 0.107 | -0.035 | 0.245 | 0.118 | -0.024 | 0.255 | -0.068 | -0.208 | 0.074 | -0.084 | -0.223 | 0.058 |
| Positive | New Friend | Emotional Significance | 0.280 | 0.144 | 0.406 | 0.296 | 0.161 | 0.420 | -0.086 | -0.225 | 0.056 | -0.130 | -0.267 | 0.012 |
| Positive | New Friend | Control | 0.121 | -0.021 | 0.258 | 0.106 | -0.036 | 0.244 | 0.126 | -0.016 | 0.263 | 0.112 | -0.030 | 0.250 |
| Positive | New Friend | Extraordinariness | -0.098 | -0.236 | 0.044 | -0.121 | -0.258 | 0.021 | 0.150 | 0.009 | 0.286 | 0.166 | 0.025 | 0.301 |
| Positive | New Friend | Impact | 0.109 | -0.033 | 0.247 | 0.129 | -0.013 | 0.266 | -0.132 | -0.269 | 0.010 | -0.149 | -0.285 | -0.008 |
| Positive | New Friend | Predictability | 0.032 | -0.110 | 0.173 | 0.036 | -0.106 | 0.177 | -0.028 | -0.169 | 0.114 | -0.033 | -0.174 | 0.109 |
| Positive | New Friend | Social Status | 0.104 | -0.038 | 0.242 | 0.104 | -0.038 | 0.242 | 0.010 | -0.132 | 0.151 | -0.004 | -0.146 | 0.138 |
| Positive | New Friend | Valence | -0.070 | -0.210 | 0.072 | -0.055 | -0.195 | 0.087 | -0.119 | -0.256 | 0.023 | -0.110 | -0.248 | 0.032 |
| Positive | Parenthood | Challenge | 0.069 | -0.073 | 0.209 | 0.076 | -0.066 | 0.215 | -0.118 | -0.255 | 0.024 | -0.122 | -0.259 | 0.020 |
| Positive | Parenthood | Worldview | 0.020 | -0.122 | 0.161 | 0.024 | -0.118 | 0.165 | -0.062 | -0.202 | 0.080 | -0.063 | -0.203 | 0.079 |
| Positive | Parenthood | Emotional Significance | 0.017 | -0.125 | 0.158 | 0.023 | -0.119 | 0.164 | -0.094 | -0.233 | 0.048 | -0.095 | -0.233 | 0.047 |
| Positive | Parenthood | Control | -0.066 | -0.206 | 0.076 | -0.064 | -0.204 | 0.078 | -0.050 | -0.190 | 0.092 | -0.047 | -0.187 | 0.095 |
| Positive | Parenthood | Extraordinariness | 0.018 | -0.124 | 0.159 | 0.014 | -0.128 | 0.155 | 0.076 | -0.066 | 0.215 | 0.075 | -0.067 | 0.214 |
| Positive | Parenthood | Impact | 0.074 | -0.068 | 0.213 | 0.081 | -0.061 | 0.220 | -0.119 | -0.256 | 0.023 | -0.124 | -0.261 | 0.018 |
| Positive | Parenthood | Predictability | 0.099 | -0.043 | 0.237 | 0.094 | -0.048 | 0.233 | 0.106 | -0.036 | 0.244 | 0.101 | -0.041 | 0.239 |
| Positive | Parenthood | Social Status | 0.180 | 0.039 | 0.314 | 0.186 | 0.046 | 0.319 | -0.094 | -0.233 | 0.048 | -0.106 | -0.244 | 0.036 |
| Positive | Parenthood | Valence | -0.094 | -0.233 | 0.048 | -0.090 | -0.229 | 0.052 | -0.096 | -0.234 | 0.046 | -0.091 | -0.230 | 0.051 |
| Positive | Workforce | Challenge | 0.127 | -0.015 | 0.264 | 0.132 | -0.010 | 0.269 | -0.073 | -0.212 | 0.069 | -0.082 | -0.221 | 0.060 |
| Positive | Workforce | Worldview | 0.134 | -0.008 | 0.270 | 0.143 | 0.001 | 0.279 | -0.104 | -0.242 | 0.038 | -0.115 | -0.252 | 0.027 |
| Positive | Workforce | Emotional Significance | 0.128 | -0.014 | 0.265 | 0.125 | -0.017 | 0.262 | 0.053 | -0.089 | 0.193 | 0.045 | -0.097 | 0.185 |
| Positive | Workforce | Control | 0.077 | -0.065 | 0.216 | 0.071 | -0.071 | 0.210 | 0.095 | -0.047 | 0.233 | 0.090 | -0.052 | 0.229 |
| Positive | Workforce | Extraordinariness | -0.061 | -0.201 | 0.081 | -0.069 | -0.209 | 0.073 | 0.101 | -0.041 | 0.239 | 0.106 | -0.036 | 0.244 |
| Positive | Workforce | Impact | 0.157 | 0.016 | 0.292 | 0.151 | 0.010 | 0.286 | 0.113 | -0.029 | 0.251 | 0.103 | -0.039 | 0.241 |
| Positive | Workforce | Predictability | 0.160 | 0.019 | 0.295 | 0.154 | 0.013 | 0.289 | 0.095 | -0.047 | 0.233 | 0.085 | -0.057 | 0.224 |
| Positive | Workforce | Social Status | 0.073 | -0.069 | 0.212 | 0.073 | -0.069 | 0.212 | 0.005 | -0.137 | 0.147 | 0.000 | -0.142 | 0.142 |
| Positive | Workforce | Valence | -0.140 | -0.276 | 0.002 | -0.135 | -0.271 | 0.007 | -0.079 | -0.218 | 0.063 | -0.070 | -0.210 | 0.072 |
| Negative | Crime | Challenge | 0.104 | -0.038 | 0.242 | 0.103 | -0.039 | 0.241 | -0.102 | -0.240 | 0.040 | -0.100 | -0.238 | 0.042 |
| Negative | Crime | Worldview | 0.059 | -0.083 | 0.199 | 0.058 | -0.084 | 0.198 | -0.082 | -0.221 | 0.060 | -0.081 | -0.220 | 0.061 |
| Negative | Crime | Emotional Significance | 0.150 | 0.009 | 0.286 | 0.151 | 0.010 | 0.286 | -0.128 | -0.265 | 0.014 | -0.125 | -0.262 | 0.017 |
| Negative | Crime | Control | 0.013 | -0.129 | 0.154 | 0.015 | -0.127 | 0.156 | 0.019 | -0.123 | 0.160 | 0.020 | -0.122 | 0.161 |
| Negative | Crime | Extraordinariness | -0.045 | -0.185 | 0.097 | -0.039 | -0.180 | 0.103 | -0.009 | -0.150 | 0.133 | -0.008 | -0.149 | 0.134 |
| Negative | Crime | Impact | 0.142 | 0.000 | 0.278 | 0.137 | -0.005 | 0.273 | -0.088 | -0.227 | 0.054 | -0.088 | -0.227 | 0.054 |
| Negative | Crime | Predictability | 0.032 | -0.110 | 0.173 | 0.030 | -0.112 | 0.171 | -0.105 | -0.243 | 0.037 | -0.104 | -0.242 | 0.038 |
| Negative | Crime | Social Status | 0.169 | 0.028 | 0.303 | 0.166 | 0.025 | 0.301 | -0.023 | -0.164 | 0.119 | -0.022 | -0.163 | 0.120 |
| Negative | Crime | Valence | 0.003 | -0.139 | 0.145 | 0.006 | -0.136 | 0.147 | 0.143 | 0.001 | 0.279 | 0.143 | 0.001 | 0.279 |
| Negative | Disaster | Challenge | 0.111 | -0.031 | 0.249 | 0.150 | 0.009 | 0.286 | -0.171 | -0.305 | -0.030 | -0.198 | -0.330 | -0.058 |
| Negative | Disaster | Worldview | 0.054 | -0.088 | 0.194 | 0.062 | -0.080 | 0.202 | -0.031 | -0.172 | 0.111 | -0.043 | -0.183 | 0.099 |
| Negative | Disaster | Emotional Significance | 0.064 | -0.078 | 0.204 | 0.104 | -0.038 | 0.242 | -0.180 | -0.314 | -0.039 | -0.197 | -0.329 | -0.057 |
| Negative | Disaster | Control | -0.080 | -0.219 | 0.062 | -0.076 | -0.215 | 0.066 | -0.028 | -0.169 | 0.114 | -0.012 | -0.153 | 0.130 |
| Negative | Disaster | Extraordinariness | 0.121 | -0.021 | 0.258 | 0.090 | -0.052 | 0.229 | 0.174 | 0.033 | 0.308 | 0.154 | 0.013 | 0.289 |
| Negative | Disaster | Impact | 0.198 | 0.058 | 0.330 | 0.246 | 0.108 | 0.375 | -0.196 | -0.328 | -0.056 | -0.245 | -0.374 | -0.107 |
| Negative | Disaster | Predictability | 0.152 | 0.011 | 0.287 | 0.210 | 0.070 | 0.341 | -0.241 | -0.370 | -0.103 | -0.280 | -0.406 | -0.144 |
| Negative | Disaster | Social Status | -0.092 | -0.231 | 0.050 | -0.112 | -0.250 | 0.030 | 0.092 | -0.050 | 0.231 | 0.112 | -0.030 | 0.250 |
| Negative | Disaster | Valence | -0.087 | -0.226 | 0.055 | -0.116 | -0.253 | 0.026 | 0.129 | -0.013 | 0.266 | 0.150 | 0.009 | 0.286 |
| Negative | Divorced | Challenge | 0.044 | -0.098 | 0.184 | 0.067 | -0.075 | 0.207 | -0.135 | -0.271 | 0.007 | -0.144 | -0.280 | -0.002 |
| Negative | Divorced | Worldview | 0.083 | -0.059 | 0.222 | 0.110 | -0.032 | 0.248 | -0.154 | -0.289 | -0.013 | -0.170 | -0.304 | -0.029 |
| Negative | Divorced | Emotional Significance | 0.154 | 0.013 | 0.289 | 0.172 | 0.031 | 0.306 | -0.098 | -0.236 | 0.044 | -0.126 | -0.263 | 0.016 |
| Negative | Divorced | Control | 0.028 | -0.114 | 0.169 | 0.034 | -0.108 | 0.175 | -0.040 | -0.181 | 0.102 | -0.045 | -0.185 | 0.097 |
| Negative | Divorced | Extraordinariness | -0.051 | -0.191 | 0.091 | -0.058 | -0.198 | 0.084 | 0.041 | -0.101 | 0.182 | 0.049 | -0.093 | 0.189 |
| Negative | Divorced | Impact | 0.178 | 0.037 | 0.312 | 0.198 | 0.058 | 0.330 | -0.101 | -0.239 | 0.041 | -0.133 | -0.270 | 0.009 |
| Negative | Divorced | Predictability | -0.093 | -0.232 | 0.049 | -0.078 | -0.217 | 0.064 | -0.103 | -0.241 | 0.039 | -0.090 | -0.229 | 0.052 |
| Negative | Divorced | Social Status | 0.021 | -0.121 | 0.162 | 0.013 | -0.129 | 0.154 | 0.054 | -0.088 | 0.194 | 0.051 | -0.091 | 0.191 |
| Negative | Divorced | Valence | -0.091 | -0.230 | 0.051 | -0.104 | -0.242 | 0.038 | 0.075 | -0.067 | 0.214 | 0.091 | -0.051 | 0.230 |
| Negative | Falling Out | Challenge | 0.211 | 0.072 | 0.342 | 0.216 | 0.077 | 0.347 | -0.095 | -0.233 | 0.047 | -0.107 | -0.245 | 0.035 |
| Negative | Falling Out | Worldview | 0.225 | 0.086 | 0.355 | 0.227 | 0.088 | 0.357 | -0.044 | -0.184 | 0.098 | -0.055 | -0.195 | 0.087 |
| Negative | Falling Out | Emotional Significance | 0.183 | 0.042 | 0.316 | 0.190 | 0.050 | 0.323 | -0.126 | -0.263 | 0.016 | -0.136 | -0.272 | 0.006 |
| Negative | Falling Out | Control | 0.021 | -0.121 | 0.162 | 0.021 | -0.121 | 0.162 | -0.012 | -0.153 | 0.130 | -0.013 | -0.154 | 0.129 |
| Negative | Falling Out | Extraordinariness | -0.080 | -0.219 | 0.062 | -0.089 | -0.228 | 0.053 | 0.183 | 0.042 | 0.316 | 0.187 | 0.047 | 0.320 |
| Negative | Falling Out | Impact | 0.204 | 0.064 | 0.336 | 0.214 | 0.075 | 0.345 | -0.156 | -0.291 | -0.015 | -0.168 | -0.302 | -0.027 |
| Negative | Falling Out | Predictability | 0.081 | -0.061 | 0.220 | 0.085 | -0.057 | 0.224 | -0.072 | -0.211 | 0.070 | -0.076 | -0.215 | 0.066 |
| Negative | Falling Out | Social Status | 0.218 | 0.079 | 0.349 | 0.220 | 0.081 | 0.351 | -0.034 | -0.175 | 0.108 | -0.045 | -0.185 | 0.097 |
| Negative | Falling Out | Valence | 0.022 | -0.120 | 0.163 | 0.018 | -0.124 | 0.159 | 0.095 | -0.047 | 0.233 | 0.094 | -0.048 | 0.233 |
| Negative | Family Died | Challenge | 0.064 | -0.078 | 0.204 | 0.074 | -0.068 | 0.213 | -0.130 | -0.267 | 0.012 | -0.135 | -0.271 | 0.007 |
| Negative | Family Died | Worldview | 0.118 | -0.024 | 0.255 | 0.123 | -0.019 | 0.260 | -0.066 | -0.206 | 0.076 | -0.075 | -0.214 | 0.067 |
| Negative | Family Died | Emotional Significance | 0.061 | -0.081 | 0.201 | 0.067 | -0.075 | 0.207 | -0.074 | -0.213 | 0.068 | -0.078 | -0.217 | 0.064 |
| Negative | Family Died | Control | 0.086 | -0.056 | 0.225 | 0.081 | -0.061 | 0.220 | 0.085 | -0.057 | 0.224 | 0.080 | -0.062 | 0.219 |
| Negative | Family Died | Extraordinariness | -0.088 | -0.227 | 0.054 | -0.097 | -0.235 | 0.045 | 0.103 | -0.039 | 0.241 | 0.111 | -0.031 | 0.249 |
| Negative | Family Died | Impact | 0.140 | -0.002 | 0.276 | 0.146 | 0.004 | 0.282 | -0.069 | -0.209 | 0.073 | -0.081 | -0.220 | 0.061 |
| Negative | Family Died | Predictability | 0.096 | -0.046 | 0.234 | 0.099 | -0.043 | 0.237 | -0.043 | -0.183 | 0.099 | -0.051 | -0.191 | 0.091 |
| Negative | Family Died | Social Status | 0.041 | -0.101 | 0.182 | 0.037 | -0.105 | 0.178 | 0.059 | -0.083 | 0.199 | 0.056 | -0.086 | 0.196 |
| Negative | Family Died | Valence | -0.037 | -0.178 | 0.105 | -0.044 | -0.184 | 0.098 | 0.086 | -0.056 | 0.225 | 0.089 | -0.053 | 0.228 |
| Negative | Family Jailed | Challenge | 0.045 | -0.097 | 0.185 | 0.084 | -0.058 | 0.223 | -0.223 | -0.353 | -0.084 | -0.234 | -0.364 | -0.096 |
| Negative | Family Jailed | Worldview | -0.011 | -0.152 | 0.131 | 0.019 | -0.123 | 0.160 | -0.179 | -0.313 | -0.038 | -0.180 | -0.314 | -0.039 |
| Negative | Family Jailed | Emotional Significance | -0.004 | -0.146 | 0.138 | 0.028 | -0.114 | 0.169 | -0.195 | -0.328 | -0.055 | -0.197 | -0.329 | -0.057 |
| Negative | Family Jailed | Control | -0.081 | -0.220 | 0.061 | -0.067 | -0.207 | 0.075 | -0.098 | -0.236 | 0.044 | -0.086 | -0.225 | 0.056 |
| Negative | Family Jailed | Extraordinariness | -0.001 | -0.143 | 0.141 | 0.023 | -0.119 | 0.164 | -0.139 | -0.275 | 0.003 | -0.141 | -0.277 | 0.001 |
| Negative | Family Jailed | Impact | 0.048 | -0.094 | 0.188 | 0.075 | -0.067 | 0.214 | -0.158 | -0.293 | -0.017 | -0.168 | -0.302 | -0.027 |
| Negative | Family Jailed | Predictability | -0.054 | -0.194 | 0.088 | -0.037 | -0.178 | 0.105 | -0.110 | -0.248 | 0.032 | -0.102 | -0.240 | 0.040 |
| Negative | Family Jailed | Social Status | -0.010 | -0.151 | 0.132 | -0.007 | -0.148 | 0.135 | -0.017 | -0.158 | 0.125 | -0.016 | -0.157 | 0.126 |
| Negative | Family Jailed | Valence | 0.036 | -0.106 | 0.177 | 0.002 | -0.140 | 0.144 | 0.210 | 0.070 | 0.341 | 0.207 | 0.067 | 0.339 |
| Negative | Fired | Challenge | 0.151 | 0.010 | 0.286 | 0.176 | 0.035 | 0.310 | -0.089 | -0.228 | 0.053 | -0.127 | -0.264 | 0.015 |
| Negative | Fired | Worldview | 0.010 | -0.132 | 0.151 | 0.027 | -0.115 | 0.168 | -0.080 | -0.219 | 0.062 | -0.082 | -0.221 | 0.060 |
| Negative | Fired | Emotional Significance | 0.135 | -0.007 | 0.271 | 0.160 | 0.019 | 0.295 | -0.096 | -0.234 | 0.046 | -0.130 | -0.267 | 0.012 |
| Negative | Fired | Control | -0.019 | -0.160 | 0.123 | -0.012 | -0.153 | 0.130 | -0.036 | -0.177 | 0.106 | -0.032 | -0.173 | 0.110 |
| Negative | Fired | Extraordinariness | -0.013 | -0.154 | 0.129 | -0.003 | -0.145 | 0.139 | -0.050 | -0.190 | 0.092 | -0.047 | -0.187 | 0.095 |
| Negative | Fired | Impact | 0.151 | 0.010 | 0.286 | 0.171 | 0.030 | 0.305 | -0.067 | -0.207 | 0.075 | -0.104 | -0.242 | 0.038 |
| Negative | Fired | Predictability | 0.151 | 0.010 | 0.286 | 0.157 | 0.016 | 0.292 | -0.011 | -0.152 | 0.131 | -0.045 | -0.185 | 0.097 |
| Negative | Fired | Social Status | 0.067 | -0.075 | 0.207 | 0.057 | -0.085 | 0.197 | 0.043 | -0.099 | 0.183 | 0.031 | -0.111 | 0.172 |
| Negative | Fired | Valence | -0.145 | -0.281 | -0.003 | -0.165 | -0.300 | -0.024 | 0.076 | -0.066 | 0.215 | 0.111 | -0.031 | 0.249 |
| Negative | Friend Died | Challenge | 0.290 | 0.155 | 0.415 | 0.320 | 0.187 | 0.442 | -0.077 | -0.216 | 0.065 | -0.160 | -0.295 | -0.019 |
| Negative | Friend Died | Worldview | 0.075 | -0.067 | 0.214 | 0.109 | -0.033 | 0.247 | -0.119 | -0.256 | 0.023 | -0.142 | -0.278 | 0.000 |
| Negative | Friend Died | Emotional Significance | 0.135 | -0.007 | 0.271 | 0.191 | 0.051 | 0.324 | -0.188 | -0.321 | -0.048 | -0.231 | -0.361 | -0.092 |
| Negative | Friend Died | Control | -0.046 | -0.186 | 0.096 | -0.070 | -0.210 | 0.072 | 0.088 | -0.054 | 0.227 | 0.103 | -0.039 | 0.241 |
| Negative | Friend Died | Extraordinariness | -0.146 | -0.282 | -0.004 | -0.132 | -0.269 | 0.010 | -0.077 | -0.216 | 0.065 | -0.042 | -0.183 | 0.100 |
| Negative | Friend Died | Impact | 0.240 | 0.102 | 0.369 | 0.252 | 0.114 | 0.380 | -0.018 | -0.159 | 0.124 | -0.082 | -0.221 | 0.060 |
| Negative | Friend Died | Predictability | 0.112 | -0.030 | 0.250 | 0.130 | -0.012 | 0.267 | -0.056 | -0.196 | 0.086 | -0.087 | -0.226 | 0.055 |
| Negative | Friend Died | Social Status | -0.014 | -0.155 | 0.128 | -0.019 | -0.160 | 0.123 | 0.019 | -0.123 | 0.160 | 0.023 | -0.119 | 0.164 |
| Negative | Friend Died | Valence | -0.151 | -0.286 | -0.010 | -0.188 | -0.321 | -0.048 | 0.121 | -0.021 | 0.258 | 0.165 | 0.024 | 0.300 |
| Negative | Illness | Challenge | 0.115 | -0.027 | 0.252 | 0.136 | -0.006 | 0.272 | -0.132 | -0.269 | 0.010 | -0.150 | -0.286 | -0.009 |
| Negative | Illness | Worldview | 0.209 | 0.069 | 0.341 | 0.220 | 0.081 | 0.351 | -0.066 | -0.206 | 0.076 | -0.098 | -0.236 | 0.044 |
| Negative | Illness | Emotional Significance | 0.166 | 0.025 | 0.301 | 0.206 | 0.066 | 0.338 | -0.240 | -0.369 | -0.102 | -0.269 | -0.396 | -0.132 |
| Negative | Illness | Control | -0.094 | -0.233 | 0.048 | -0.096 | -0.234 | 0.046 | 0.009 | -0.133 | 0.150 | 0.022 | -0.120 | 0.163 |
| Negative | Illness | Extraordinariness | -0.003 | -0.145 | 0.139 | 0.019 | -0.123 | 0.160 | -0.160 | -0.295 | -0.019 | -0.161 | -0.296 | -0.020 |
| Negative | Illness | Impact | 0.178 | 0.037 | 0.312 | 0.207 | 0.067 | 0.339 | -0.178 | -0.312 | -0.037 | -0.207 | -0.339 | -0.067 |
| Negative | Illness | Predictability | 0.066 | -0.076 | 0.206 | 0.078 | -0.064 | 0.217 | -0.078 | -0.217 | 0.064 | -0.088 | -0.227 | 0.054 |
| Negative | Illness | Social Status | -0.017 | -0.158 | 0.125 | -0.010 | -0.151 | 0.132 | -0.052 | -0.192 | 0.090 | -0.050 | -0.190 | 0.092 |
| Negative | Illness | Valence | -0.175 | -0.309 | -0.034 | -0.191 | -0.324 | -0.051 | 0.099 | -0.043 | 0.237 | 0.126 | -0.016 | 0.263 |
| Negative | Jailed | Challenge | 0.157 | 0.016 | 0.292 | 0.173 | 0.032 | 0.307 | -0.127 | -0.264 | 0.015 | -0.146 | -0.282 | -0.004 |
| Negative | Jailed | Worldview | 0.091 | -0.051 | 0.230 | 0.102 | -0.040 | 0.240 | -0.099 | -0.237 | 0.043 | -0.110 | -0.248 | 0.032 |
| Negative | Jailed | Emotional Significance | 0.103 | -0.039 | 0.241 | 0.106 | -0.036 | 0.244 | -0.020 | -0.161 | 0.122 | -0.032 | -0.173 | 0.110 |
| Negative | Jailed | Control | -0.031 | -0.172 | 0.111 | -0.045 | -0.185 | 0.097 | 0.126 | -0.016 | 0.263 | 0.130 | -0.012 | 0.267 |
| Negative | Jailed | Extraordinariness | 0.069 | -0.073 | 0.209 | 0.084 | -0.058 | 0.223 | -0.132 | -0.269 | 0.010 | -0.140 | -0.276 | 0.002 |
| Negative | Jailed | Impact | 0.071 | -0.071 | 0.210 | 0.078 | -0.064 | 0.217 | -0.062 | -0.202 | 0.080 | -0.070 | -0.210 | 0.072 |
| Negative | Jailed | Predictability | -0.076 | -0.215 | 0.066 | -0.080 | -0.219 | 0.062 | 0.034 | -0.108 | 0.175 | 0.042 | -0.100 | 0.183 |
| Negative | Jailed | Social Status | 0.098 | -0.044 | 0.236 | 0.120 | -0.022 | 0.257 | -0.186 | -0.319 | -0.046 | -0.198 | -0.330 | -0.058 |
| Negative | Jailed | Valence | -0.118 | -0.255 | 0.024 | -0.133 | -0.270 | 0.009 | 0.124 | -0.018 | 0.261 | 0.139 | -0.003 | 0.275 |
| Negative | Laid Off | Challenge | 0.218 | 0.079 | 0.349 | 0.226 | 0.087 | 0.356 | -0.064 | -0.204 | 0.078 | -0.087 | -0.226 | 0.055 |
| Negative | Laid Off | Worldview | 0.157 | 0.016 | 0.292 | 0.168 | 0.027 | 0.302 | -0.100 | -0.238 | 0.042 | -0.117 | -0.254 | 0.025 |
| Negative | Laid Off | Emotional Significance | 0.163 | 0.022 | 0.298 | 0.189 | 0.049 | 0.322 | -0.220 | -0.351 | -0.081 | -0.239 | -0.368 | -0.101 |
| Negative | Laid Off | Control | 0.053 | -0.089 | 0.193 | 0.061 | -0.081 | 0.201 | -0.076 | -0.215 | 0.066 | -0.081 | -0.220 | 0.061 |
| Negative | Laid Off | Extraordinariness | -0.095 | -0.233 | 0.047 | -0.094 | -0.233 | 0.048 | -0.016 | -0.157 | 0.126 | -0.007 | -0.148 | 0.135 |
| Negative | Laid Off | Impact | 0.209 | 0.069 | 0.341 | 0.225 | 0.086 | 0.355 | -0.134 | -0.270 | 0.008 | -0.158 | -0.293 | -0.017 |
| Negative | Laid Off | Predictability | 0.200 | 0.060 | 0.332 | 0.199 | 0.059 | 0.331 | 0.026 | -0.116 | 0.167 | 0.007 | -0.135 | 0.148 |
| Negative | Laid Off | Social Status | 0.210 | 0.070 | 0.341 | 0.216 | 0.077 | 0.347 | -0.048 | -0.188 | 0.094 | -0.070 | -0.210 | 0.072 |
| Negative | Laid Off | Valence | -0.178 | -0.312 | -0.037 | -0.194 | -0.327 | -0.054 | 0.146 | 0.004 | 0.282 | 0.166 | 0.025 | 0.301 |
| Negative | Parent Died | Challenge | 0.077 | -0.065 | 0.216 | 0.120 | -0.022 | 0.257 | -0.184 | -0.317 | -0.044 | -0.205 | -0.337 | -0.065 |
| Negative | Parent Died | Worldview | -0.015 | -0.156 | 0.127 | 0.021 | -0.121 | 0.162 | -0.169 | -0.303 | -0.028 | -0.170 | -0.304 | -0.029 |
| Negative | Parent Died | Emotional Significance | -0.062 | -0.202 | 0.080 | -0.013 | -0.154 | 0.129 | -0.241 | -0.370 | -0.103 | -0.233 | -0.363 | -0.095 |
| Negative | Parent Died | Control | 0.052 | -0.090 | 0.192 | 0.030 | -0.112 | 0.171 | 0.110 | -0.032 | 0.248 | 0.102 | -0.040 | 0.240 |
| Negative | Parent Died | Extraordinariness | 0.081 | -0.061 | 0.220 | 0.067 | -0.075 | 0.207 | 0.074 | -0.068 | 0.213 | 0.059 | -0.083 | 0.199 |
| Negative | Parent Died | Impact | -0.023 | -0.164 | 0.119 | -0.011 | -0.152 | 0.131 | -0.055 | -0.195 | 0.087 | -0.052 | -0.192 | 0.090 |
| Negative | Parent Died | Predictability | 0.054 | -0.088 | 0.194 | 0.072 | -0.070 | 0.211 | -0.076 | -0.215 | 0.066 | -0.090 | -0.229 | 0.052 |
| Negative | Parent Died | Social Status | 0.111 | -0.031 | 0.249 | 0.108 | -0.034 | 0.246 | 0.026 | -0.116 | 0.167 | 0.003 | -0.139 | 0.145 |
| Negative | Parent Died | Valence | -0.012 | -0.153 | 0.130 | -0.034 | -0.175 | 0.108 | 0.099 | -0.043 | 0.237 | 0.104 | -0.038 | 0.242 |
| Negative | Partner Died | Challenge | -0.001 | -0.143 | 0.141 | 0.007 | -0.135 | 0.148 | -0.140 | -0.276 | 0.002 | -0.140 | -0.276 | 0.002 |
| Negative | Partner Died | Worldview | -0.017 | -0.158 | 0.125 | -0.014 | -0.155 | 0.128 | -0.055 | -0.195 | 0.087 | -0.054 | -0.194 | 0.088 |
| Negative | Partner Died | Emotional Significance | 0.044 | -0.098 | 0.184 | 0.049 | -0.093 | 0.189 | -0.099 | -0.237 | 0.043 | -0.101 | -0.239 | 0.041 |
| Negative | Partner Died | Control | 0.100 | -0.042 | 0.238 | 0.099 | -0.043 | 0.237 | 0.027 | -0.115 | 0.168 | 0.022 | -0.120 | 0.163 |
| Negative | Partner Died | Extraordinariness | 0.002 | -0.140 | 0.144 | 0.003 | -0.139 | 0.145 | -0.021 | -0.162 | 0.121 | -0.021 | -0.162 | 0.121 |
| Negative | Partner Died | Impact | 0.010 | -0.132 | 0.151 | 0.016 | -0.126 | 0.157 | -0.113 | -0.251 | 0.029 | -0.114 | -0.252 | 0.028 |
| Negative | Partner Died | Predictability | 0.190 | 0.050 | 0.323 | 0.197 | 0.057 | 0.329 | -0.108 | -0.246 | 0.034 | -0.120 | -0.257 | 0.022 |
| Negative | Partner Died | Social Status | 0.121 | -0.021 | 0.258 | 0.119 | -0.023 | 0.256 | 0.043 | -0.099 | 0.183 | 0.037 | -0.105 | 0.178 |
| Negative | Partner Died | Valence | 0.077 | -0.065 | 0.216 | 0.073 | -0.069 | 0.212 | 0.065 | -0.077 | 0.205 | 0.062 | -0.080 | 0.202 |
