# Supplementary material for: Attachment-related perceptions of life events
Source: PLoS One. 2026 Jan 6;21(1):e0340082. doi: 10.1371/journal.pone.0340082 (PMC12774378; doi:10.1371/journal.pone.0340082)
Supplement: Attachment — Submitted filename: ResponseToReviewers101225.docx [file pone.0340082.s004.docx]

**Response to Reviewers**

We would like to thank the editor and reviewers for their thoughtful comments on the manuscript. We very much appreciate the constructive feedback and believe that the manuscript has improved significantly as a result of their suggestions. Below, we report how each of the reviewer issues was addressed and the corresponding changes to the manuscript (we also submitted a tracked changes version of the paper). The reviewer comments are non-bolded, and our responses are bolded.

**Reviewer #1**

Dear Authors,

Thank you for the opportunity to review this interesting and well-executed manuscript. You have undertaken a commendable study on a theoretically rich and important topic, exploring how attachment orientations shape the perceptual lens through which life events are viewed. The application of Luhmann et al.’s taxonomy is a particular strength. I have several suggestions that I believe could help strengthen the theoretical contribution and clarity of the paper.

Major Comments:

1. Deepening the Theoretical Framework: The “Why” Question

The introduction and discussion would benefit greatly from moving beyond describing the differences to explaining them. The findings are a perfect springboard to discuss the underlying motivational and cognitive mechanisms of attachment strategies.

Attachment Anxiety: The results suggest anxious individuals perceive events as highly impactful, negative, and identity-altering. This should be explicitly framed as a consequence of their hyperactivating strategies. Their chronic need for proximity and fear of abandonment creates a cognitive-affective framework where events are intensely scrutinized for their relational meaning and potential threat. This aligns with their need to create a coherent narrative (often negative) that justifies their anxiety and motivates their search for reassurance.

Attachment Avoidance: The dismissive perceptions of avoidant individuals are a textbook example of deactivating strategies. Their defensive minimization of event significance, emotional impact, and potential for change is a core strategy to maintain independence and suppress attachment-related distress. This isn't just a "difference"; it's a strategic downregulation of the perceived importance of events to avoid the vulnerability that comes with acknowledging their impact.

I strongly recommend restructuring the discussion around these theoretical frameworks (hyperactivation/deactivation) to answer the "why" question more compellingly.

**Thanks for recommending this revised structure. We agree that it provides a closer introduction to the empirical tests we conducted. We have now significantly changed the introduction—removing the large amount of text on attachment-related changes (which we now realize was a bit tangential). Rather, we took the opportunity, as the reviewer suggested, to dedicate more space to the cognitive and motivational tendencies of people high in attachment anxiety and avoidance. These revisions can be seen on lns 42-125 and are reproduced below:**

**An individual’s attachment orientation is how they generally approach close relationships and is thought to be formed by early interactions with caregivers, though experiences with relationships later in life likely also affect attachment orientations [1, 2, 10-15]. Variation in attachment orientation is characterized by how people vary on two dimensions—attachment anxiety and attachment avoidance [16]. Attachment anxiety is characterized by fear of rejection and a preoccupation with seeking reassurance from close others [17]. Attachment avoidance is characterized by a discomfort with closeness and dependence [18]. Since its extension from childhood to adult romantic and platonic relationships, attachment theory has served as an organizing framework for characterizing thoughts, feelings, and behavior in the context of close relationships [19, 20]. People low in both attachment anxiety and attachment avoidance are considered to be relatively securely attached.**

**Although there are normative shifts in attachment orientations across the lifespan, attachment orientations are generally considered stable individual difference characteristics [12, 21-23]. One of the reasons why attachment orientations are stable is because of the cognitive and motivational mechanisms that reify one’s existing “working model” of attachment to promote stability [4, 11, 24, 25]. In other words, like other psychological characteristics, attachment orientations guide the situations people find themselves in, how they interpret those situations, and can also fundamentally change aspects of those situations [26, 27]. People higher in attachment anxiety and avoidance tend to interpret situations in ways that are consistent with what they think about themselves and other people [24, 28-31].**

**For example, people higher in attachment anxiety tend to engage in hyperactivation of the attachment system as a way of interpreting the world and regulating their emotions and behavior. Attachment-related threats typically trigger efforts to increase proximity to attachment figures and to secure support from them [32, 33]. Collectively, these strategies are meant to prevent abandonment [34]. People high in attachment anxiety tend to hold negative perceptions of themselves, think their self-worth is conditional, and have heightened rejection sensitivity to what they feel are potentially unavailable attachment figures [35-37]. Hyperactivation of the attachment system is accompanied by a hyperfixation on the availability of close others, intense scrutiny over support provision, selective attention toward rejection-related cues, and constant threat-monitoring [38-41]. Among people higher in attachment anxiety, this hyperactivation is often made manifest in intense feelings of jealousy (a vigilance to the attention and commitment of others), maladaptive attributions of ambiguous behavior (assuming others are inconsistently reliable as a way of monitoring availability), and otherwise perceiving the world in a more negative light, as this is consistent with their internal way of thinking about the world [30, 42, 43]. Although these attributional and motivational tendencies make sense in light of their attachment histories and working models, this hyperactivation of the attachment system often results in greater error in threat detection, an ironic *undermining* of relational commitment from partners, rumination and catastrophizing, and an inaccurate warping of relational circumstances—all correlates of less satisfying interpersonal relationships [17, 44-47]. As a result, when confronted with either an ambiguous life event or situation, people high in attachment anxiety would likely perceive it to be more negative for them, their relationships, and their future.**

**In contrast, people higher in attachment avoidance engage in chronic attempts to *deactivate* the attachment system. When attachment needs are triggered, people high in attachment avoidance typically engage in strategies designed to suppress dependency, dampen emotional activation, and maintain distance from attachment figures [18, 34, 48, 49]. Collectively, these strategies are meant to prevent vulnerability and protect autonomy rather than pursue closeness [32, 50]. People high in attachment avoidance tend to hold a negative perception of close others—often believing others are unreliable, intrusive, or emotionally overwhelming—while maintaining a compensatory positive self-view centered on independence and self-sufficiency [35, 51, 52]. Deactivation of the attachment system is accompanied by selective attention away from emotional cues, minimizing the personal relevance of relational events, and the suppression or dismissal of attachment-related thoughts and feelings [53-55]. Among people higher in attachment avoidance, this deactivation often manifests in emotional disengagement, reluctance to self-disclose, downplaying relational difficulties, and attributing ambiguous situations in ways that preserve psychological distance and self-reliance [40, 56, 57]. Although these tendencies serve an immediate regulatory function by reducing perceived relational threat, they may also result in reduced emotional awareness, impaired intimacy, and difficulty acknowledging support when it is offered—patterns that predict lower relationship satisfaction and reduced partner responsiveness over time [58-61]. As a result, when confronted with an ambiguous event or situation, people high in attachment avoidance would likely perceive it as less personally or relationally relevant and respond with emotional disengagement rather than threat sensitivity.**

**In these ways, people high in attachment anxiety or avoidance have several cognitive and motivational tendencies that shape how they perceive themselves, others, and the world. And these tendencies—and the perceptions they shape—likely explain why people’s attachment orientations are stable, even when disruptive and significant life shocks or events happen to them [62-64], even if some relational contexts might alter these enduring processes [5, 60, 65]. But the study of life events and their impact on attachment changes has been limited in several ways [8]. As an illustration of just how powerful these mindsets are, Fraley and colleagues [7] followed over 4,000 people over a mean time frame of 2 years to examine how their attachment orientations changed before-to-after 25 different life events that encapsulated both relational and non-relational contexts. Although many life events had an initial association with changes in attachment orientations, very few had *enduring* effects. Importantly, this work rarely examines people’s qualitative assessments of life events—a potential avenue through which attachment orientations might exert their influence. In the current study, we examined how people high in attachment anxiety and avoidance perceive life events as an illustrative case to assess their cognitive and motivational tendencies—tendencies that explain sources of anxiety and avoidance and why they might be durable to changes in situations and life events. But comprehensive models for conceptualizing the range of perceptions people have in response to events are rare. However, researchers have recently developed a taxonomy for characterizing people’s perceptions of life events.**

**We also revised one of the early parts of the Discussion to discuss how the personality-change-perceptions results are consistent with the aforementioned motivational and cognitive mechanisms outlined in the intro. This addition can be found in lines 446-486 and is reproduced here:**

**The present findings extend adult attachment theory by demonstrating that people’s perceptions of life events map meaningfully onto the cognitive and motivational tendencies that underlie attachment anxiety and avoidance [20, 32, 36, 82]. Rather than reflecting random variation or general negativity, these patterns appear to be consistent with stable working models that guide how individuals attend to, interpret, and emotionally respond to potentially meaningful events. In line with theoretical frameworks emphasizing biased cognitive processing in insecure attachment, our results suggest that people higher in attachment anxiety and avoidance do not simply perceive hypothetical life events differently, but they perceive and evaluate those circumstances in ways that reinforce their existing relational beliefs. Thus, rather than serving as corrective experiences, life events may function psychologically as confirmatory evidence that maintains—rather than disrupts—attachment orientations [26, 28, 30, 31, 83-85].**

**Individuals higher in attachment anxiety appeared particularly likely to interpret life events as impactful, emotionally significant, and potentially destabilizing, consistent with hyperactivating strategies that prioritize vigilance, emotional amplification, and threat sensitivity. Their greater belief that events could meaningfully alter personality may reflect an underlying sense of vulnerability and uncertainty about relational stability [17, 47]. Given their tendency to scrutinize relational cues and interpret ambiguous information as threatening or rejection-relevant [30], anxious individuals may perceive life events—especially those involving transition or uncertainty—as opportunities for potential loss, change, or validation of their feared expectations [76]. This aligns with work showing that individuals high in anxiety engage in rumination, catastrophizing, and selective attention toward negative relational cues, which may contribute to the perception that life experiences have powerful and lasting consequences [86]. Taken together, the current findings suggest that anxious individuals’ heightened emotional and cognitive engagement with life events may increase the likelihood that such events shape their relational beliefs over time.**

**Attachment avoidance was more robustly associated with personality change perceptions. Conversely, though, people higher in attachment avoidance perceived nearly all life events as low in personal relevance and unlikely to influence their personality. This pattern is consistent with deactivating strategies aimed at minimizing reliance on others, suppressing emotional engagement, and maintaining psychological distance [87-89]. Avoidant individuals’ tendency to downplay the relational and emotional significance of events may serve as a defensive mechanism that protects their preferred autonomy and reduces exposure to vulnerability. Prior research demonstrates that avoidant adults often exhibit attentional disengagement, reduced emotional recall, and reinterpretation of relational experiences as inconsequential [44, 54, 55]. All of these behavioral tendencies may contribute to the belief that external circumstances—including potentially disruptive or meaningful ones—pose little threat to their self-concept. In this way, avoidance may buffer individuals from perceived relational instability but, paradoxically, may also prevent them from using life experiences as opportunities for growth or relational recalibration.**

**Finally, there are a few additional sentences throughout the Discussion that tie the findings back to these recommended sections.**

2. Nuancing the Link to Personality (Big Five)

The authors briefly mention the work on Big Five traits but do not fully integrate this into their narrative. This is a missed opportunity. Given the well-established correlations (Attachment Anxiety ~ Neuroticism; Attachment Anxiety and Attachment Avoidance ~ low Agreeableness), the current findings could be partly explained by these broader traits.

For instance, is the heightened negative perception in anxious individuals merely a reflection of their neuroticism? The discussion should explicitly address this potential confound and argue for the unique contribution of attachment working models over and above the Big Five. A control analysis (e.g., partial correlations controlling for Neuroticism) would be a powerful addition, though even a theoretical discussion of this point is necessary.

**We acknowledge that links between attachment orientations and Big Five personality traits could have been made more explicit. We have now expanded the section on Big Five personality traits and life event perceptions. We now more deliberately describe the overlap between attachment and personality, including the trait-specific associations the reviewer recommends. However, we also provide some additional discussion about the unique contribution that attachment orientations provide for life and relational outcomes, over-and-above the Big Five personality traits. This addition can be found in lines 138-155 and is reproduced below:**

**There have also been links established between the Big Five personality traits and these life event perceptions [71]. Reviewing associations with the Big Five personality traits is valuable given their empirical overlap with attachment orientations and because they share some of the same attributional and evaluative tendencies mentioned above [72]. For example, highly agreeable people—a characteristic shared by those lower attachment avoidance—thought social life events (e.g., divorce, falling out with a friend, marriage, making a new close friend, becoming a parent) were more controllable and less harmful to their social status. Likewise, people high in neuroticism—a characteristic shared by those higher in attachment anxiety—thought life events overall were more challenging, emotionally significant, impactful, and had negative effects on their social status. Some of these findings are intuitive, but not all personality traits were related to life event perceptions in intuitive ways (e.g., conscientiousness was not particularly related to work or school life event perceptions). One reasonable critique of the attachment literature is that attachment-related differences might be reducible to personality-related differences (e.g., the heightened negative perception of people high in attachment anxiety might be merely a reflection of their high levels of neuroticism). Worth noting, associations between attachment orientations and Big Five personality traits are modest, such that they are not empirically redundant. And importantly, attachment orientations provide additional explanatory power for life and relational outcomes over-and-above Big Five personality traits [41, 72, 73].**

3. Interpretation of Weak Effects and Gender Imbalance

The authors correctly note the correlations are weak. This warrants a more critical discussion. Do these small effects suggest that while statistically discernible in a large sample, attachment orientation is a relatively minor player in event perception compared to other factors (e.g., the specific event itself, broader personality)? The practical significance of these findings needs to be addressed.

**We agree that it is important to make this discussion (of small correlations) more explicit in the manuscript. We have now added caveats in several places, including the Results (lns 319-320: “Associations between attachment orientations and personality change perceptions were relatively small.”; lns 319-320: “Associations between attachment orientations and life event perceptions are reported in Table 2 and were generally small.”; lns 334-335: “Associations between attachment orientations and perceptions of positive life events were generally weak and inconsistent”, and lns 367-368: “In summary, while attachment anxiety and avoidance were associated with select perceptions of both positive and negative life events, these patterns were inconsistent and generally small in magnitude. The findings suggest that attachment orientations may shape people’s expectations about life events in limited and context-dependent ways, particularly when considering hypothetical scenarios.”**

**We also added a stand-alone paragraph to the Discussion where we explicitly discuss how small the correlations are, what can be reasonably gleaned from such small effect sizes, and how they might be obscuring heterogeneity (e.g., that attachment orientations might matter more for some people than others, or as life events accumulate). This addition can be found in lines 428-445 and is reproduced here:**

**To contextualize our results, the associations reported here were very small in magnitude, even though they were statistically significant. This pattern likely stems from our large sample of participants who provided multiple life event perceptions, as smaller samples often overestimate effects sizes [81]. Are these small-but-significant associations of *practical* significance though? When asking about event perceptions in a hypothetical and decontextualized way, there are likely many contributing factors that affect the magnitude of these associations. Although our focus was on attachment orientations, for which we found some patterns consistent with previous work, the particular impact of these factors in more specific contexts is something our study did not examine. At such a broad level, one could characterize the results of the current study has small and, in some cases, negligible. However, it is possible that the influence of attachment orientations might be enhanced or reduced depending on other aspects of the context (e.g., real/actual life events, other characteristics of the life even or participants). Thus, although the associations found here are relatively small, their impact across people and settings might be more heterogeneous than what is reflected here. In other words, attachment orientations might be more influential in describing life event perceptions for some people than others (or as life events or social context experiences accumulate). Below, we contextualize our results in the existing literature. However, we encourage caution in evaluating these results given the relatively small effect sizes.**

**Finally, we retained the text in the Discussion that the reviewer was referring to (in the Limitations and Future Directions section). Thus, we try to make explicit to readers how small the effects are in the Results and at the start/end of the Discussion and talk about the practical relevance of the results (and the start/end of the Discussion). Finally, we also inserted a sentence early in the Discussion acknowledging these small associations (ln 414-415: “Although the effects were generally small, a few consistent patterns emerged.”).**

3.5. The sample is 74% women. Given established gender differences in attachment (higher anxiety in women, higher avoidance in men), this imbalance likely skewed the data. The authors must discuss how this may have affected the results. For example, did the preponderance of women inflate the mean levels of anxiety and the effects related to it? Would the avoidance-related effects be stronger in a more gender-balanced sample? This is a significant limitation that should be discussed in depth.

**We have now added a stand-alone paragraph discussing the gender and age limitations of the sample. This addition can be found in lines 584-602 and is reproduced below:**

**Finally, our sample was comprised primarily of women and younger adults. It is possible that the gender and age composition of the sample might have altered the magnitude of some of our associations. Women tend to report higher attachment anxiety, and men tend to report higher avoidance [100], although the magnitude of these differences can be smaller among middle-age and older adults [6]. As a result, our sample was likely biased toward characterizing young women’s experiences in particular. And men and women often have unique responses to relational circumstances [101, 102]. Thus, the large number of women might have yielded a sample higher in attachment anxiety (and lower in attachment avoidance), on average. This bias was likely compounded by the primary inclusion of younger adults. There are normative shifts in attachment anxiety across the lifespan, such that attachment anxiety is higher among younger adults, although age differences in attachment avoidance are a bit more mixed [3, 6, 21, 91, 103-105]. Likewise, there are also age-related differences in event perceptions, both in terms of which events are more common and how they are evaluated according to Luhmann et al.’s taxonomy [106]. Specifically, health-related events are more common among older adults and education-related events are more common among younger adults. Further, young adults perceived life events more positively and as more likely to change their worldviews. Perceptions were often more intense if experienced at a non-normative age. Altogether, the evidence that attachment and event perceptions vary across age and gender suggests that future research should strive to collect more age- and gender-balanced samples moving forward.**

4. Terminology and Clarity

Please use consistent terminology throughout the manuscript. Phrases like "insecurely attached people," "people high in attachment avoidance," and "avoidantly attached people" are used interchangeably. For clarity and precision, I recommend standardizing to the dimensional language: "individuals high in attachment anxiety" and "individuals high in attachment avoidance."

**We agree that it is important to maintain consistency in our description of people higher in attachment anxiety and avoidance. We have now gone through the manuscript and adopted this dimensional language (which is also more precise). We now refer to these people as individuals/people high(er) in attachment anxiety or avoidance throughout.**

5. Implications Section

The discussion would be strengthened by a dedicated paragraph on the implications of these findings. For example:

Clinical Implications: Understanding these perceptual biases can inform therapy. Helping an anxious client recognize their tendency to catastrophize events, or an avoidant client to acknowledge their minimizing strategies, could be a fruitful therapeutic target.

Theoretical Implications: The findings support a model where attachment styles are not just outcomes but active filters that shape how experiences are construed, which in turn may reinforce the existing attachment style, creating a self-perpetuating cycle.

**We have now added a stand-alone section articulating the clinical and theoretical implications of the current work. These additions can be found in lines 603--639 and are reproduced here:**

**The current work has important clinical and theoretical implications. For example, many existing clinical psychology frameworks suggest that enduring vulnerabilities and diatheses, when paired with particular external circumstances, result in the development of interpersonal difficulties [39, 107-109]. Perceiving an event as more negatively valanced, challenging, extraordinary, and negatively affecting one’s social status are associated with declines in life satisfaction after that life event [70]. Attachment orientations could be considered a pre-existing characteristic that could potentially shape how people perceive life events or how these perceptions translate to adaptation following the life event. For example, although the associations were small, people high in attachment anxiety perceived life events as more challenging, negatively valanced, and more likely to negatively impact their social status. In a therapeutic context, this information could be valuable in assessing how people might navigate important life shocks and to develop intervention plans catered to the concerns of people high in attachment anxiety. People high in attachment avoidance tended to minimize the emotional significance of life events. Although this might initially be useful in reducing stress and anxiety, such distancing might inadvertently prevent adaptive processing of life events’ impact on people or stymie therapeutic efforts to change the everyday behavior of people high in attachment avoidance.**

**Most broadly, from a theoretical perspective, situating attachment orientations in an emergent event perceptions classification system provides a unifying framework for understanding attachment-related differences in responses to life events. Overall, actual life events exert little effect on attachment orientation [7]. The lack of effects seen in previous research might be due in part to the missing context of how people were perceiving and evaluating those life events. For example, people in attachment anxiety have a propensity to catastrophize which was illustrated by their perception of hypothetical events as more challenging, impactful, and able to negatively affect their social status. Evaluating life events in these ways might reify the internal working models and attributional tendencies of people high in attachment anxiety—one of the suggested reasons why psychological characteristics are so stable over time [26]. The same can be said for attachment avoidance. By downplaying the impact of life events, people high in attachment avoidance might be reifying their defensive perceptions of the world. But this consistency likely comes at a cost: by not properly evaluating life events, particularly relational ones, people high in attachment avoidance might be less likely to alter their behavior to salvage a relationship, reflect on a disruptive experience, or seek out support from others. In both of these cases, attachment orientations filter one’s experience and reinforce the behavior consistent with their attachment orientation. Having a broader understanding of life event perceptions reveals the exact ways that these filters might exert their influence in the lives of people high in attachment anxiety and avoidance.**

Conclusion:

This is a valuable study with solid methodology that addresses an important question. With revisions that deepen the theoretical explanation, acknowledge limitations more critically, and clarify the narrative, it has the potential to be a strong contribution to the literature.

**Thank you for your helpful comments on our manuscript—we believe the paper is now greatly improved!**

**Reviewer #2**

The paper addresses an important and timely question: how individuals with different attachment orientations perceive and evaluate significant life events. The topic is highly relevant for understanding both personality development and subjective interpretations of change-inducing experiences. The study is well-powered and offers a broad empirical dataset. Nevertheless, I have several major concerns regarding both the framing of the paper and the analytic strategy.

1. According to the title, the abstract, and the main body of empirical work, the central focus of this article is how attachment orientations influence perceptions of life events — whether events are viewed as challenging, impactful, emotionally significant, unpredictable, negative, or likely to induce personality change. From this perspective, it is somewhat surprising that the theoretical section devotes substantial space to discussing whether attachment orientations themselves change over time. This issue, while relevant to the general literature on attachment, does not appear to be directly aligned with the empirical aims of the present study. Instead, the introduction would have benefited from a more focused review of how attachment orientations may shape the perception and appraisal of life events.

**We agree that the large focus on attachment-related changes across the lifespan undermined our goals of linking attachment orientations to life event perceptions. Guided by your (and R1 #1’s) comments, we substantially reduced the sections on attachment-related changes (which now constitute a minor section). Instead, we focused more on the cognitive and motivational tendencies of people high in attachment anxiety and avoidance (and correlates of those tendencies). We now feel that this revised introduction more seamlessly links motivational tendencies and attributions to how people might perceive life events differently (in ways that reify these tendencies). This new section can be found in lines 42-125 and is reproduced below (an accompanying section was also written for the Discussion; see R1#1 for more details):**

**An individual’s attachment orientation is how they generally approach close relationships and is thought to be formed by early interactions with caregivers, though experiences with relationships later in life likely also affect attachment orientations [1, 2, 10-15]. Variation in attachment orientation is characterized by how people vary on two dimensions—attachment anxiety and attachment avoidance [16]. Attachment anxiety is characterized by fear of rejection and a preoccupation with seeking reassurance from close others [17]. Attachment avoidance is characterized by a discomfort with closeness and dependence [18]. Since its extension from childhood to adult romantic and platonic relationships, attachment theory has served as an organizing framework for characterizing thoughts, feelings, and behavior in the context of close relationships [19, 20]. People low in both attachment anxiety and attachment avoidance are considered to be relatively securely attached.**

**Although there are normative shifts in attachment orientations across the lifespan, attachment orientations are generally considered stable individual difference characteristics [12, 21-23]. One of the reasons why attachment orientations are stable is because of the cognitive and motivational mechanisms that reify one’s existing “working model” of attachment to promote stability [4, 11, 24, 25]. In other words, like other psychological characteristics, attachment orientations guide the situations people find themselves in, how they interpret those situations, and can also fundamentally change aspects of those situations [26, 27]. People higher in attachment anxiety and avoidance tend to interpret situations in ways that are consistent with what they think about themselves and other people [24, 28-31].**

**For example, people higher in attachment anxiety tend to engage in hyperactivation of the attachment system as a way of interpreting the world and regulating their emotions and behavior. Attachment-related threats typically trigger efforts to increase proximity to attachment figures and to secure support from them [32, 33]. Collectively, these strategies are meant to prevent abandonment [34]. People high in attachment anxiety tend to hold negative perceptions of themselves, think their self-worth is conditional, and have heightened rejection sensitivity to what they feel are potentially unavailable attachment figures [35-37]. Hyperactivation of the attachment system is accompanied by a hyperfixation on the availability of close others, intense scrutiny over support provision, selective attention toward rejection-related cues, and constant threat-monitoring [38-41]. Among people higher in attachment anxiety, this hyperactivation is often made manifest in intense feelings of jealousy (a vigilance to the attention and commitment of others), maladaptive attributions of ambiguous behavior (assuming others are inconsistently reliable as a way of monitoring availability), and otherwise perceiving the world in a more negative light, as this is consistent with their internal way of thinking about the world [30, 42, 43]. Although these attributional and motivational tendencies make sense in light of their attachment histories and working models, this hyperactivation of the attachment system often results in greater error in threat detection, an ironic *undermining* of relational commitment from partners, rumination and catastrophizing, and an inaccurate warping of relational circumstances—all correlates of less satisfying interpersonal relationships [17, 44-47]. As a result, when confronted with either an ambiguous life event or situation, people high in attachment anxiety would likely perceive it to be more negative for them, their relationships, and their future.**

**In contrast, people higher in attachment avoidance engage in chronic attempts to *deactivate* the attachment system. When attachment needs are triggered, people high in attachment avoidance typically engage in strategies designed to suppress dependency, dampen emotional activation, and maintain distance from attachment figures [18, 34, 48, 49]. Collectively, these strategies are meant to prevent vulnerability and protect autonomy rather than pursue closeness [32, 50]. People high in attachment avoidance tend to hold a negative perception of close others—often believing others are unreliable, intrusive, or emotionally overwhelming—while maintaining a compensatory positive self-view centered on independence and self-sufficiency [35, 51, 52]. Deactivation of the attachment system is accompanied by selective attention away from emotional cues, minimizing the personal relevance of relational events, and the suppression or dismissal of attachment-related thoughts and feelings [53-55]. Among people higher in attachment avoidance, this deactivation often manifests in emotional disengagement, reluctance to self-disclose, downplaying relational difficulties, and attributing ambiguous situations in ways that preserve psychological distance and self-reliance [40, 56, 57]. Although these tendencies serve an immediate regulatory function by reducing perceived relational threat, they may also result in reduced emotional awareness, impaired intimacy, and difficulty acknowledging support when it is offered—patterns that predict lower relationship satisfaction and reduced partner responsiveness over time [58-61]. As a result, when confronted with an ambiguous event or situation, people high in attachment avoidance would likely perceive it as less personally or relationally relevant and respond with emotional disengagement rather than threat sensitivity.**

**In these ways, people high in attachment anxiety or avoidance have several cognitive and motivational tendencies that shape how they perceive themselves, others, and the world. And these tendencies—and the perceptions they shape—likely explain why people’s attachment orientations are stable, even when disruptive and significant life shocks or events happen to them [62-64], even if some relational contexts might alter these enduring processes [5, 60, 65]. But the study of life events and their impact on attachment changes has been limited in several ways [8]. As an illustration of just how powerful these mindsets are, Fraley and colleagues [7] followed over 4,000 people over a mean time frame of 2 years to examine how their attachment orientations changed before-to-after 25 different life events that encapsulated both relational and non-relational contexts. Although many life events had an initial association with changes in attachment orientations, very few had *enduring* effects. Importantly, this work rarely examines people’s qualitative assessments of life events—a potential avenue through which attachment orientations might exert their influence. In the current study, we examined how people high in attachment anxiety and avoidance perceive life events as an illustrative case to assess their cognitive and motivational tendencies—tendencies that explain sources of anxiety and avoidance and why they might be durable to changes in situations and life events. But comprehensive models for conceptualizing the range of perceptions people have in response to events are rare. However, researchers have recently developed a taxonomy for characterizing people’s perceptions of life events.**

2. In reporting the results, the authors analyze correlations separately for attachment anxiety and attachment avoidance. However, prior research consistently shows that these two dimensions are positively intercorrelated. As a consequence, some of the reported associations may be confounded by this interrelation. A more appropriate analytic strategy would be to present partial correlations controlling for the other attachment dimension, or, preferably, to use regression models in which both attachment anxiety and avoidance are entered simultaneously as predictors of each perception rating. This would provide a clearer and more valid picture of the unique contributions of each attachment dimension.

**Thanks for recommending this approach. Because attachment anxiety and avoidance were significantly correlated (*r* = .13, *p* < .001), it is important to account for this covariation in the analyses. We have now computed partial correlations and provided them in the main text (i.e., Tables 1 and 2) and a Supplementary Table X. The associations were largely the same, although when they did change, they often *grew* in magnitude. Some associations became significant after accounting for the other dimension; however, these were among the smallest effects in the Results and they tended to hover around *p* = .05. Given the comparable size (and the fact that those near-*p*=.05 associations would be unlikely to survive a correction), we largely interpreted the associations that were significant as a raw/bivariate and partial correlation. These changes can be found in the tables and this description of the results and approach can be found in lines 283-295 and is reproduced here:**

**Because attachment anxiety and avoidance were positively associated with each other, we also computed partial correlations in which we estimated the association between one attachment orientation (i.e., attachment anxiety) and a life event/perception while controlling for the other attachment orientation. These partial correlations are presented alongside the raw/bivariate correlations. For the most part, the results were highly similar across the two correlations. The associations were typically larger after accounting for the other attachment orientation. There were a few scenarios in which a null bivariate association became significant after controlling for the other attachment orientation. For the Results, we largely discussed the associations that were consistently significant using both approaches. We did this because of the high similarity between the two, the interpretational ambiguity of one attachment orientation being “purged” or unconfounded from the other, and because the few associations that were significant in one approach but not the other were typically very small and unlikely to survive even a modest alpha correction. Nevertheless, they are reported in the tables for transparency.**

3. The manuscript highlights that individuals higher in attachment anxiety tend to perceive certain events (e.g., divorce, job loss, relocation) as more likely to induce personality change. However, the actual correlation coefficients for these examples are on the order of r = .05–.06. Such effect sizes explain only a negligible fraction of variance, and the statistical significance is a function of the very large sample size rather than substantive explanatory power. By contrast, the associations with attachment avoidance, while still modest (rarely exceeding r = .15), are consistently stronger than those observed for attachment anxiety. Yet the discussion does not adequately acknowledge this pattern, giving the impression that anxiety plays the dominant role when the data suggest the opposite.

**We agree that it is important to contextualize the size of the associations in our study. As seen in our response to R1 #3, we have now added text in several places that speak to the small/negligible associations and their implications for evaluating their practical significance (in the Results and in multiple places in the Discussion).**

**However, we agree that it is important to highlight the results for attachment avoidance (reported in Table 2) were more robust than those for anxiety. We have now added this observation to the Results just before describing them, in lines 326-328 (“The pattern of associations with personality change perceptions and attachment avoidance were much larger than those seen for attachment anxiety, although still modest in the absolute sense.”). In the text, we also added text to note this in a few places, including lns 415-418 (“First, people high in attachment avoidance largely thought that their personalities would be less affected by both positive and negative life events; people high in attachment anxiety thought their personalities would change more dramatically, although attachment anxiety was not as robustly related to personality change perceptions.”) and ln 473 (“Attachment avoidance was more robustly associated with personality change perceptions.”).**

4. In the section Attachment Orientation and Event Characteristics, the authors pool all events and compute correlations between attachment dimensions and event ratings across approximately 3,800–3,900 observations. However, these observations are not independent, as each participant provided ratings for multiple events. Attachment anxiety and avoidance are measured at the person level, whereas challenge, worldview change, emotional significance, and other characteristics are measured at the event level, nested within participants. Simple correlations therefore ignore the non-independence of the data and likely inflate the effective sample size. A more appropriate analytic approach would be multilevel modeling (e.g., mixed-effects regression), with events nested within persons, which would allow for proper estimation of effects while accounting for shared variance at the participant level.

**Thanks for bringing this up. We actually did this analysis in the original manuscript—it was reported in Footnote #4 and in Supplementary Table 1. However, we understand that readers might miss that we did these analyses, so we moved the footnote to the main text in lines 348-352; this text is reproduced below:**

**Because participants provided multiple event perception ratings, there is a non-independence issue in the data. We ran follow-up tests predicting each event perception from anxiety or avoidance in the context of a multi-level model (with random intercepts for participants). The results were identical in interpretation as reported in Table 2. The results of these multi-level models can be found in Supplementary Table S1.**

5. In the section Attachment Orientations and Event Characteristics for Specific Life Events, the authors again present separate correlations for attachment anxiety and attachment avoidance, further split into positive and negative life events. This approach suffers from the same limitation noted above: it does not control for the correlation between anxiety and avoidance, making it unclear which associations are unique to each attachment dimension. As before, a regression approach including both dimensions simultaneously would provide more interpretable and theoretically meaningful results.

**We have now added columns to Supplementary Table 2 which presents the partial correlations. Like the previous analyses, the patterns of correlations were highly similar (i.e., the magnitudes of the bivariate and partial correlations were high for both attachment anxiety (r = .99) and avoidance (r = .99). Of the 360 possible associations tested, only 20 changed significance levels when the other dimension was partialled out (5.56%; 3 became non-significant; 17 became significant), and most of these associations were close to p = .05 (and would be unlikely to survive an alpha correction, which we note in the text). This change can be seen in Supplementary Table S2 and a brief note has been added to the manuscript in lines 362-366, which is reproduced here:**

**For this information in table form, for both bivariate and partial correlations, see Supplementary Table S2. Comparing the bivariate and partial correlations revealed that only about 5% of the associations changed in significance level (3 went from significant to non-significant; 17 went from non-significant to significant), although these were typically just below *p* = .05 and would be unlikely to survive an alpha correction; see the supplement for full details.**

**Reviewer #3**

Thank you for allowing me to review your study. I found it quite engaging and valuable, especially since it tackles an important gap in attachment research by exploring the link between attachment orientations and perceptions of life events. I identified some theoretical, methodological, and interpretive issues that somewhat limit its overall contribution.

1. How did you approach investigating attachment in relation to how people perceive subjective events, beyond studies that only examine the occurrence of events?

Authors should also emphasize the importance of integrating attachment theory with emerging event classification systems.

**Thanks for encouraging us to more deliberately yoke the discussion of attachment orientations to the study of hypothetical life events. We addressed this comment in three ways. First, we have now added a stand-alone paragraph in the introduction where we set-up why examining links between attachment orientations and this emerging classification system was important. This addition can be found in lines 156--176 and is reproduced here:**

**To date, however, there has not been an examination of how individuals high in attachment anxiety and avoidance differ in their perceptions of life events using this expanded taxonomy. However, there are some reasons why attachment orientations might have implications for how people evaluate hypothetical life events as specified by the Luhmann et al. taxonomy. For example, individuals high in attachment anxiety tend to excessively seek reassurance from others [17]. As a result, individuals high in attachment anxiety might evaluate ostensibly negative relational events (e.g., dissolutions, falling out with a friend) as more negatively valenced, more impactful, and more challenging. Individuals high in attachment avoidance tend to employ deactivating strategies to minimize distress [32, 48]. As a result, individuals high in attachment avoidance might evaluate ostensibly distressing life events (e.g., losing a family member or friend), being the victim of a crime) as less emotionally significant and less likely to impact their worldview—as acknowledging the impact of life events might be accompanied with distress. These differences would be consistent with the appraisal and attributional tendencies of individuals high in attachment anxiety and avoidance discussed above. Our study moves beyond evaluating whether life events are more or less likely to happen to individuals high in attachment anxiety and avoidance. Rather, we examined associations between attachment orientations and this emerging classification system to clarify attachment-related differences in qualitative perceptions of hypothetical life events. Doing so situates attachment orientations as a potential predictor of Luhmann et al.’s taxonomy and can encourage future research examining adaptation to these life events among individuals high in attachment anxiety or avoidance.**

**Second, we added an expanded Implications section in the Discussion. In this section, we talk about the importance of assessing perceptions of hypothetical events for both therapeutic/clinical and theoretical reasons. This addition can be found in lines 603-639 and is reproduced here:**

**The current work has important clinical and theoretical implications. For example, many existing clinical psychology frameworks suggest that enduring vulnerabilities and diatheses, when paired with particular external circumstances, result in the development of interpersonal difficulties [39, 107-109]. Perceiving an event as more negatively valanced, challenging, extraordinary, and negatively affecting one’s social status are associated with declines in life satisfaction after that life event [70]. Attachment orientations could be considered a pre-existing characteristic that could potentially shape how people perceive life events or how these perceptions translate to adaptation following the life event. For example, although the associations were small, people high in attachment anxiety perceived life events as more challenging, negatively valanced, and more likely to negatively impact their social status. In a therapeutic context, this information could be valuable in assessing how people might navigate important life shocks and to develop intervention plans catered to the concerns of people high in attachment anxiety. People high in attachment avoidance tended to minimize the emotional significance of life events. Although this might initially be useful in reducing stress and anxiety, such distancing might inadvertently prevent adaptive processing of life events’ impact on people or stymie therapeutic efforts to change the everyday behavior of people high in attachment avoidance.**

**Most broadly, from a theoretical perspective, situating attachment orientations in an emergent event perceptions classification system provides a unifying framework for understanding attachment-related differences in responses to life events. Overall, actual life events exert little effect on attachment orientation [7]. The lack of effects seen in previous research might be due in part to the missing context of how people were perceiving and evaluating those life events. For example, people in attachment anxiety have a propensity to catastrophize which was illustrated by their perception of hypothetical events as more challenging, impactful, and able to negatively affect their social status. Evaluating life events in these ways might reify the internal working models and attributional tendencies of people high in attachment anxiety—one of the suggested reasons why psychological characteristics are so stable over time [26]. The same can be said for attachment avoidance. By downplaying the impact of life events, people high in attachment avoidance might be reifying their defensive perceptions of the world. But this consistency likely comes at a cost: by not properly evaluating life events, particularly relational ones, people high in attachment avoidance might be less likely to alter their behavior to salvage a relationship, reflect on a disruptive experience, or seek out support from others. In both of these cases, attachment orientations filter one’s experience and reinforce the behavior consistent with their attachment orientation. Having a broader understanding of life event perceptions reveals the exact ways that these filters might exert their influence in the lives of people high in attachment anxiety and avoidance.**

**Finally, in responding to another reviewer, we significantly revised the first part of the intro to more deliberately focus on attachment-related differences in appraisal processes, which provided a more seamless transition to the study of hypothetical life events (rather than focusing on attachment-related change); see R1 #1.**

2. From a methodological standpoint, relying on U.S. undergraduates (average age = 19.6, 74% women) restricts the generalizability of the findings; while the WEIRD bias is acknowledged, it remains a major concern.

**We agree that our sample composition and methodological design are significantly limited. To address these points, we have now added an expanded paragraph explaining the implications of our relatively young sample (and also the gender imbalance as well). This addition can be found in lines 584-602 and is reproduced here:**

**Finally, our sample was comprised primarily of women and younger adults. It is possible that the gender and age composition of the sample might have altered the magnitude of some of our associations. Women tend to report higher attachment anxiety, and men tend to report higher avoidance [100], although the magnitude of these differences can be smaller among middle-age and older adults [6]. As a result, our sample was likely biased toward characterizing young women’s experiences in particular. And men and women often have unique responses to relational circumstances [101, 102]. Thus, the large number of women might have yielded a sample higher in attachment anxiety (and lower in attachment avoidance), on average. This bias was likely compounded by the primary inclusion of younger adults. There are normative shifts in attachment anxiety across the lifespan, such that attachment anxiety is higher among younger adults, although age differences in attachment avoidance are a bit more mixed [3, 6, 21, 91, 103-105]. Likewise, there are also age-related differences in event perceptions, both in terms of which events are more common and how they are evaluated according to Luhmann et al.’s taxonomy [106]. Specifically, health-related events are more common among older adults and education-related events are more common among younger adults. Further, young adults perceived life events more positively and as more likely to change their worldviews. Perceptions were often more intense if experienced at a non-normative age. Altogether, the evidence that attachment and event perceptions vary across age and gender suggests that future research should strive to collect more age- and gender-balanced samples moving forward.**

**We also agree that the WEIRD limitation could have used a bit more explication. We have now expanded that limitation from the original manuscript to more reflexively discuss its implications. This addition can be seen in lines 561-572 and is reproduced here:**

**Second, our participants were from one particular culture (i.e., the United States). Most psychological research is conducted in predominantly WEIRD (Western, educated, industrialized, rich, democratic) countries—a considerable limitation to the current work. There are certainly studies that have examined variation in attachment across cultures [91-95]. Given that demographic variation in attachment orientations differ across regions and cultures, it seems likely that how people perceive and evaluate life events also differs [93, 96], for which there is evidence [97, 98]. However, the study of life events, both with respect to attachment and psychological characteristics more broadly, has been predominantly done in WEIRD cultures [8, 99]. It is an important future direction to elevate the study of life events (and their perceptions), attachment, and their intersection into the cross-cultural space. Just as attachment differs across cultures [93], event perceptions and their impact on attachment changes across the lifespan might also vary.**

3. Another issue is the cross–sectional design based on hypothetical scenarios. I believe that perceptions of imaginary events may not accurately reflect real experiences, and making causal claims about how life events influence attachment is questionable.

**Although we discussed some of these limitations in the original manuscript, we agree that it is important to deliberately discuss how the cross-sectional design and hypothetical scenarios were limiting factors of our study. We have now expanded this paragraph, which can be found in lines 542-560 and is reproduced below:**

**First, we relied on cross-sectional data and hypothetical life events. Appropriately examining how life events change a person’s attachment orientation requires longitudinal data, including assessments of attachment orientations before and after an event [e.g., 7]. Unfortunately, data sets that have multiple assessments of attachment orientations over time often do not have comprehensive assessments of life events or their perceived characteristics (nor do studies of life events [and their characteristics] measure attachment very often). In other words, researchers might have information on whether a life event happened and occasionally multiple assessments of attachment. Still, they lack description and specificity of how life events were perceived or affected people. Our study suggests that there were attachment-related differences in how people perceived hypothetical life events, suggesting that these perceptions might have differential effects on how attachment changes when people actually experience events. However, there is no guarantee that these hypothetical perceptions extend to real events. In this way, our study was limited in its ability to make causal claims for characterizing how attachment orientations would be related to evaluations of actual life events and experiences, or how how people might adapt to actual life events that happen to them. Future research should more deliberately integrate information about how participants subjectively perceive the impact of actual—not hypothetical—life events along a broad array of indicators [9]. These studies would enable us to test whether these perceptions (and not just the presence or absence of a life event) lead to changes in attachment orientation.**

4. In terms of data analysis, some ECQ subscales (e.g., worldview, α = 0.59, and impact, α = 0.60) exhibit low reliability.

**We have now added a limitation to the paper acknowledging these low reliabilities. This addition can be found in lines 269-273 and is reproduced below:**

**Some of these subscales have relatively low reliabilities, which is likely attributable to our use of the short-form measure of the scale (i.e., two-items per dimension compromises reliability and additional items would provide greater precision). Although these reliabilities are consistent with previous research [9, 71], future research can employ the long-form versions of these instruments.**

5. Additionally, most identified relationships are very weak between the studied constructs. Although statistically significant, these limitations raise doubts about their practical significance.

**We agree that it is important to contextualize the size of the associations in our study. As seen in our response to R1 #3, we have now added text in several places that speak to the small/negligible associations and their implications for evaluating their practical significance (in the Results and in multiple places in the Discussion). Here is that response reproduced from above:**

**We agree that it is important to make this discussion (of small correlations) more explicit in the manuscript. We have now added caveats in several places, including the Results (lns 319-320: “Associations between attachment orientations and personality change perceptions were relatively small.”; lns 334-335: “Associations between attachment orientations and life event perceptions are reported in Table 2 and were generally small.”; lns 367-368: “Associations between attachment orientations and perceptions of positive life events were generally weak and inconsistent”, and lns 407-409: “In summary, while attachment anxiety and avoidance were associated with select perceptions of both positive and negative life events, these patterns were inconsistent and generally small in magnitude. The findings suggest that attachment orientations may shape people’s expectations about life events in limited and context-dependent ways, particularly when considering hypothetical scenarios.”**

**We also added a stand-alone paragraph to the Discussion where we explicitly discuss how small the correlations are, what can be reasonably gleaned from such small effect sizes, and how they might be obscuring heterogeneity (e.g., that attachment orientations might matter more for some people than others, or as life events accumulate). This addition can be found in lines 428-445 and is reproduced here:**

**To contextualize our results, the associations reported here were very small in magnitude, even though they were statistically significant. This pattern likely stems from our large sample of participants who provided multiple life event perceptions, as smaller samples often overestimate effects sizes [81]. Are these small-but-significant associations of *practical* significance though? When asking about event perceptions in a hypothetical and decontextualized way, there are likely many contributing factors that affect the magnitude of these associations. Although our focus was on attachment orientations, for which we found some patterns consistent with previous work, the particular impact of these factors in more specific contexts is something our study did not examine. At such a broad level, one could characterize the results of the current study has small and, in some cases, negligible. However, it is possible that the influence of attachment orientations might be enhanced or reduced depending on other aspects of the context (e.g., real/actual life events, other characteristics of the life even or participants). Thus, although the associations found here are relatively small, their impact across people and settings might be more heterogeneous than what is reflected here. In other words, attachment orientations might be more influential in describing life event perceptions for some people than others (or as life events or social context experiences accumulate). Below, we contextualize our results in the existing literature. However, we encourage caution in evaluating these results given the relatively small effect sizes.**

**Finally, we retained the text in the Discussion that the reviewer was referring to (in the Limitations and Future Directions section). Thus, we try to make explicit to readers how small the effects are in the Results and at the start/end of the Discussion and talk about the practical relevance of the results (and the start/end of the Discussion). Finally, we also inserted a sentence early in the Discussion acknowledging these small associations (ln 414-415: “Although the effects were generally small, a few consistent patterns emerged.”).**

6. Some conclusions, such as the resilience of avoidant individuals and the greater reactivity of anxious individuals, might overstretch the small correlational data.

**We have now substantially revised the manuscript to center discussions on how small the associations were while revising the strong claims that attachment orientations are robust predictors of these evaluations (e.g., we removed language about resilience and reactivity). These changes can be seen throughout the manuscript, and we thank the reviewer for encouraging us to more appropriately contextualize our results.**
